# Nonsteroidal anti-inflammatory drugs (NSAIDs), cyxlooxygenase-2 selective inhibitors (coxibs) and gastrointestinal harm: review of clinical trials and clinical practice

**DOI:** 10.1186/1471-2474-7-79

**Published:** 2006-10-20

**Authors:** R Andrew Moore, Sheena Derry, Ceri J Phillips, Henry J McQuay

**Affiliations:** 1 Pain Research and Nuffield Department of Anaesthetics, University of Oxford, The Churchill, Headington, Oxford, OX3 7LJ, UK; 2 School of Health Sciences, University of Wales Swansea, Singleton Park, Swansea, SA2 8PP, UK

## Abstract

**Background:**

Gastrointestinal harm, known to occur with NSAIDs, is thought to be lower with NSAID and gastroprotective agent, and with inhibitors selective to cyclooxygenase-2 (coxibs) at usual plasma concentrations. We examine competing strategies for available evidence of reduced gastrointestinal bleeding in clinical trials and combine this evidence with evidence from clinical practice on whether the strategies work in the real world, whether guidance on appropriate prescribing is followed, and whether patients adhere to the strategies.

**Methods:**

We used a series of systematic literature searches to find full publications of relevant studies for evidence about the efficacy of these different gastroprotection strategies in clinical trials, and for evidence that they worked and were adhered to in clinical practice – whether they were effective. We chose to use good quality systematic reviews and meta-analyses when they were available.

**Results:**

Evidence of efficacy of coxibs compared to NSAIDs for upper gastrointestinal bleeding was strong, with consistent reductions in events of about 50% in large randomised trials (34,460 patients), meta-analyses of randomised trials (52,474 patients), and large observational studies in clinical practice (3,093 bleeding events). Evidence on the efficacy of NSAID plus gastroprotection with acid suppressants (proton pump inhibitors, PPIs, and histamine antagonists, H2As) was based mainly on the surrogate measure of endoscopic ulcers. The limited information on damage to the bowel suggested that NSAID plus PPI was more damaging than coxibs.

Eleven observational studies studied 1.6 million patients, of whom 911,000 were NSAID users, and showed that 76% (range 65% to 90%) of patients with at least one gastrointestinal risk factor received no prescription for gastroprotective agent with an NSAID. The exception was a cohort of US veterans with previous gastrointestinal bleeding, where 75% had gastroprotection with an NSAID. When gastroprotection was prescribed, it was often described as inadequate. A single study suggested that patient adherence to prescribed gastroprotection was low.

**Conclusion:**

Evidence for efficacy of gastroprotection strategies with NSAIDs is limited. In clinical practice few patients who need gastroprotection get it, and those who get it may not take it. For coxibs, gastroprotection is inherent, although probably not complete.

## Background

Chronic pain affects one adult in five in Europe [1], limits functioning, and is an enormous problem for healthcare. Osteoarthritis, rheumatoid arthritis, and back pain have the largest negative impact on quality of life of any chronic condition (including cancer, chronic respiratory conditions, or heart disease) for people living in the community [2].

NSAIDs are effective analgesic and anti-inflammatory drugs that form the main pharmacological approach to treating various forms of pain, and particularly chronic musculoskeletal pain, but have a number of known adverse effects. NSAIDs (and aspirin) are associated with upper [3] and lower [4-6] gastrointestinal harm, acute renal failure [7,8] and congestive heart failure [9,10]. Coxibs are differentiated pharmacologically from traditional NSAIDs by inhibiting only the cyclooxygenase-2 enzyme, and clinically by lower rates of upper and lower gastrointestinal harm. All of these drugs (aspirin, NSAIDs, and coxibs) may also be associated with increased risk of cardiovascular harm, although increased cardiovascular events are not generally seen for coxibs compared with NSAIDs or placebo in studies in patients with arthritis. Meta-analyses of large numbers of patients in trials of individual coxibs [11] and all coxibs [12] found no systematic difference between coxib and NSAID. Meta-analysis of recent observational studies with 3.5 million patients showed that cardiovascular effects of some NSAIDs (particularly diclofenac) were greater than some coxibs [13]. Our views on rare but serious harm can be directed by the amount of information available.

This paper concentrates on differences between NSAIDs and coxibs for causing gastrointestinal harm. Possible strategies for reducing gastrointestinal harm from NSAIDs alone include use of coxib, NSAID plus PPI, NSAID plus H2A, or NSAID plus misoprostol. Since misoprostol is prescribed rarely in the UK [14] and elsewhere because of other gastrointestinal adverse events it causes, the competing strategies for gastroprotection are use of histamine antagonists or proton pump inhibitors with NSAID, or coxib.

The effectiveness of any strategy is the product of efficacy in clinical trials, and the usability of the strategy in clinical practice. For drugs, this means that prescribing of a medicine is appropriate, and that patients prescribed the medicine take it. Medicines not taken cannot be effective.

We examine each competing strategy in terms of available evidence for reduction of gastrointestinal bleeding in clinical studies and combine it with evidence from clinical practice on whether the strategies work in the real world, whether guidance on appropriate prescribing is followed, and whether patients are able or willing to adhere to the strategies over the longer term.

## Methods

We searched for evidence from systematic reviews, randomised trials and observational studies of clinical practice in several areas:

1. Evidence of reduction of upper and lower gastrointestinal bleeding rates with coxibs compared with non-selective NSAIDs; in particular, evidence that results obtained from clinical trials were also seen in clinical practice.

2. Evidence concerning levels of prescribing of gastroprotective strategies with non-selective NSAIDs to patients with one or more risk factors for gastrointestinal bleeding, and whether prescribing was described as appropriate against any prescribing guidance.

3. Evidence concerning adherence to prescribed gastroprotective strategies with non-selective NSAIDs.

A number of different search strategies were used to find full publications of studies relating to these outcomes. These were predominantly free-text searches of PubMed and the Cochrane Library (to December 2005), bibliographies of papers and reviews, and discussions with experts. Where evidence was available, we chose the highest level available, preferably from good quality systematic reviews and meta-analyses. The sensitivity of electronic databases for observational studies is known not to be high [15,16], so reviews and bibliographies were extensively searched for references to studies of prescribing strategies in clinical practice.

Any study that might have contributed was obtained in full and read. For inclusion, the only criterion was that of full publication; abstracts or posters were not accepted. Formal quality scoring of included studies was not considered appropriate because of the likely mix of systematic reviews, randomised trials, and observational studies. The review was considered to be a descriptive narrative review, without extensive pooling of data. No statistical methods were planned, and none used.

## Results

### Reduction of upper gastrointestinal bleeding

Table 1 summarises evidence relating to the outcomes of complicated upper gastrointestinal bleeding and/or symptomatic ulcer from three large randomised trials powered to detect these events (34,460 patients; [17-19]), and six meta-analyses of randomised double blind trials (52,474 patients in total, 44,415 of whom were not in one of the large randomised trials; [20-25]). Some of the meta-analyses include information from the large randomised trials, with some inevitable, but limited, duplication. The number of events was as low as 11 in one meta-analysis [21] and as high as 283 in one large randomised trial [19]. Whether the outcome was complicated bleeding events, symptomatic ulcers, or the combination, the rate with coxibs was consistently about half that with NSAIDs. In randomised trials, coxibs had significantly lower rates for upper gastrointestinal bleeding, symptomatic ulcers, endoscopic ulcers, anaemia, and withdrawal due to gastrointestinal symptoms (Table 2). For endoscopic ulcers, benefits for coxib over NSAID were of the same absolute magnitude (number needed to prevent 8) with or without low dose aspirin [25].

Three large observational studies [26-28] with 3,093 bleeding events compared NSAIDs and coxibs in clinical practice (Table 1). Each of these studies noted that patients receiving coxibs had more gastrointestinal risk factors than those receiving NSAIDs (channelling bias), and adjusted risk estimates for this confounding. Each of them used the outcome of hospital admission for gastrointestinal bleeding, usually upper gastrointestinal bleeding. The number of events with particular treatments could be low; only 17 admissions occurred with NSAID in one [26], and only four with coxibs in another [27]. Despite the relative paucity of events, the risk of hospital admission with coxib was about half that with NSAID. Clinical practice produced the same magnitude of reduction for coxib compared with NSAID as did clinical trials.

### Reduction of lower gastrointestinal bleeding

Lower gastrointestinal bleeding has not been extensively studied. In the 1990s, using radioactive indium-labelled white cells, it was shown that faecal excretion of white cells (a marker of intestinal inflammation) was elevated with oral NSAID. Calprotectin, a calcium binding protein found in neutrophilic granulocytes, monocytes, and macrophages, which resists faecal degradation was also used as a marker. Use of the test in 312 patients taking NSAIDs showed that 44% had raised faecal calprotectin concentrations, much the same as estimates with indium studies [29]. A retrospective analysis of a large (8,000 patient) randomised trial comparing daily rofecoxib 50 mg with naproxen 1000 mg used three markers for lower gastrointestinal bleeding (gross rectal bleeding with haemoglobin decrease of 20 g/L or admission; haemoglobin decrease, positive faecal blood, no upper gastrointestinal bleeding; admission for lower perforation, obstruction, ulceration or diverticulitis) [30]. It showed a significant reduction of these events (by about 50%) with coxib compared with NSAID, and that lower gastrointestinal bleeding was about 40% of total (upper plus lower) gastrointestinal bleeding events. A more recent randomised trial [6] compared celecoxib 400 mg daily with placebo and naproxen 1000 mg plus omeprazole 20 mg daily, in 360 healthy volunteers over two weeks. Capsule endoscopy found significantly more small bowel mucosal breaks per patient with celecoxib (0.3 breaks per patient on average) than with placebo (0.1), but many more with naproxen plus omeprazole (3.0).

Another marker of blood loss from the bowel may be anaemia. Oral diclofenac 150 mg daily produced anaemia in 10% of about 310 patients in 12 weeks [31]. Hooper et al [24] noted a significantly lower rate of anaemia with coxibs compared with NSAIDs (Table 2). A large meta-analysis of celecoxib trials using clinical trial reports to ascertain adverse events used two markers of anaemia, a haemoglobin fall of ≥ 20 g/L, or a haematocrit fall of ≥ 5% by the end of the study [25]. Celecoxib and placebo were not significantly different, while rates with celecoxib were always lower than those with NSAID, with numbers needed to prevent one case of 92 and 18 for haemoglobin and haematocrit respectively compared with NSAID.

#### NSAID plus PPI

Only three upper gastrointestinal bleeds were noted in a meta-analysis of randomised trials using NSAID plus PPI vs NSAID alone ([24]; Table 2), and only 18 symptomatic ulcers. NSAIDs plus PPI had lower rates of endoscopic ulcer and withdrawal due to gastrointestinal symptoms, though with only 48 events for the latter calculation (Table 2).

Two randomised trials [32,33] have directly compared celecoxib 200 or 400 mg daily with NSAID plus PPI (diclofenac 150 mg plus omeprazole 20 mg daily, or naproxen 750 mg plus lanzoprazole 30 mg daily). Both were conducted in patients with a previous ulcer bleed, and who needed NSAID for arthritis. After ulcer healing, patients were randomised to treatments over six months. Serious gastrointestinal complications (bleeding events) were no different for celecoxib than NSAID plus PPI (4.2% vs 6.0%; relative risk 0.7, 95% confidence interval 0.3 to 1.5). There were similar rates of discontinuations due to adverse events (4.5% vs 3.8%; relative risk 1.2, 0.5 to 2.8), but dyspepsia was significantly more common with coxib than NSAID plus PPI (15% vs 7%; relative risk 2.1, 1.3 to 3.6).

Lower gastrointestinal mucosal breaks were more common with naproxen plus omeprazole than coxib or placebo [6]. Two observational studies using capsule endoscopy also found high levels of small bowel injury taking diclofenac 150 mg plus omeprazole 40 mg daily in volunteers [34] and in patients with NSAID use for three months or more plus some form of gastroprotection [35].

#### NSAID plus H2A

The only evidence for efficacy for histamine antagonists with NSAID was for endoscopic ulcers (Table 2). There was no evidence of efficacy for histamine antagonists protecting against lower bowel injury.

### Appropriate prescribing

We found 11 studies related to the appropriateness of use of gastroprotective strategies in patients using NSAIDs [36-46] (Table 3). These studies included 1.56 million patients, of whom 911,000 were recipients of NSAIDs.

**Figure 1:**
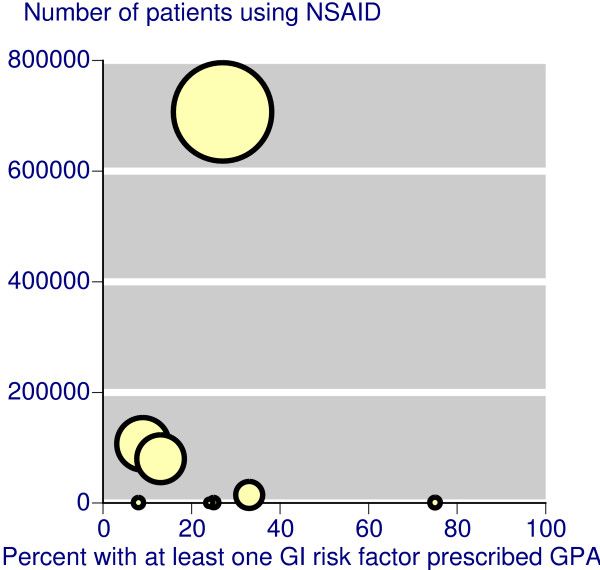
Scatter plot showing the percentage of patients with at least one gastrointestinal risk factor prescribed an NSAID plus gastroprotective agent (H2A or PPI) or a coxib. The size of the symbol is proportional to the size of the study.

Eight of the 11 studies reported that large proportions of patients with gastrointestinal risk factors (including age ≥ 65 years) were not receiving appropriate gastroprotection. In what appeared to be mainly primary care populations, non-use of gastroprotection in patients with at least one gastrointestinal risk factor was about 73% to 90% in the USA [37,44], 76% in Italy [38], 87% in Holland [39], 65% in Canada [40], and 76% in the UK [45]. A study in secondary care in the UK found no gastroprotection in 76% of patients with at least one gastrointestinal risk factor [46], but gastroprotection non-use was lower at 25% in a cohort of patients following a diagnosed ulcer or bleed [41]. Pooling these 11 studies (Figure 1), 76% of the patients with at least one gastrointestinal risk factor did not receive a prescription for a gastroprotective agent.

Four of the 11 studies made some comment on the adequacy or appropriateness of prescribing of NSAIDs and coxibs, with or without gastroprotection. It was not always clear what specific guidelines were used to judge appropriateness, and results varied greatly. Two Dutch studies [36,42] agreed on the adequacy of gastroprotection, at 55% and 65% respectively. Both defined inadequate prescribing as a lower dose H2A. In Canada prescribing was deemed appropriate in 33% of patients with no risk factors, and in 74% of those with at least one risk factor [43]. By contrast, in UK secondary care only 8% of NSAID users were deemed to have appropriate treatment [46], and of those prescribed gastroprotection, 56% were prescribed PPI, without comment on whether doses of other gastroprotective agents were effective.

Three studies [36,43,45] commented on factors associated with a higher propensity to be prescribed gastroprotection. Consistently mentioned were having two or more risk factors, older age, and history of prior bleeding.

Three further studies [47-49] (Table 1) confirmed that gastroprotection was not used in about 80% of NSAID users, while not providing information about risk factors.

### Adherence

We could find only a single study examining adherence to gastroprotection. Sturkenboom et al [36] followed 784 patients receiving PPI or H2A with NSAID. Half the users of H2A had become non-adherent after about 100 days, rising to about 70% by a year. Adherence was better with PPI, but non-adherence was about 40% by a year, and had risen to about 60% by the longest follow up of two years.

In a cohort of 711 subjects who stopped their NSAID and who were followed up for up to two years [36], about 40% had at least one additional prescription for acid suppressing medicine after stopping NSAID, and somehat more than 30% had at least two prescriptions.

## Discussion

Current UK clinical guidance on NSAID use [50] includes the following risk factors for developing an NSAID-induced gastrointestinal adverse event.

• Age of 65 years and over.

• Previous history of gastroduodenal ulcer, gastrointestinal bleeding, or gastroduodenal perforation.

• Concomitant use of medications that are known to increase the likelihood of upper-gastrointestinal adverse events (anticoagulants, aspirin, including low-dose aspirin, and corticosteroids).

• Presence of serious comorbidity, such as cardiovascular disease, renal or hepatic impairment, diabetes, or hypertension.

• Requirement for a prolonged duration of NSAID use.

• Use of the maximum recommended doses of NSAIDs.

• The presence of Helicobacter pylori infection.

• Excessive alcohol use.

• Heavy smoking.

Using these criteria, many NSAID users have a risk factor, even if it is only age. A large US study found that 42% of 707,000 NSAID users were at higher risk [44], and in a recent Italian study at least one risk factor was found in 68% of NSAID users [51]. Many different guidelines exist, and they consistently recommend use of gastroprotective agent (PPI or H2A) with NSAID, or use of coxib. All strategies include interventions that should precede oral NSAID use, such as lifestyle change, topical NSAIDs, paracetamol, and glucosamine; this study did not include any of these, as they are not conventionally implicated in gastrointestinal damage.

This review set out to examine the strength of evidence on particular gastroprotective strategies, whether guidance about strategies was implemented by prescribers, and whether patients were adherent to prescribed medicines. The evidence available is mixed: some points are addressed by large and coherent amounts of evidence, while for others evidence is either lacking or limited. An important limitation is that much of the information, especially in observational studies in clinical practice, does not differentiate between different NSAIDs or gastroprotective agents. Moreover, while some evidence is of the highest level from meta-analyses of high-quality randomised trials [52], other evidence is from observational studies, where testing of quality is more difficult. We do know, however, that when criteria of quality, validity, and size are met, observational studies produce similar results to randomised trials [53].

The evidence on efficacy in reducing upper gastrointestinal bleeding is considerable for coxibs. Three adequately-powered randomised trials, and several meta-analyses of large numbers of patients in randomised trials come to the consistent conclusion that the rate of complicated upper gastrointestinal events with coxibs is about half that with NSAIDs. Rates of symptomatic ulcers, endoscopic ulcers, and withdrawal because of gastrointestinal adverse events were all about half with coxibs compared with NSAIDs. For coxibs there is also evidence, from capsule endoscopy studies, of reduced lower bowel damage compared with NSAID plus PPI. Additionally, rates of anaemia were lower with coxibs than with NSAIDs in two large meta-analyses [24,25], using several definitions of anaemia. This represents a large body of evidence, with many events (Table 2), using clinical as well as surrogate outcomes, and supported by large observational studies of high quality.

Strategies of co-prescription of NSAID with either PPI or H2A do not have this degree of evidence to support reduction of serious gastrointestinal complications. One reason for is that this indication came considerably later than original licensing of these gastroprotective agents and the surrogate marker of endoscopic ulcers was used. Absence of large outcome trials does not mean that they do not work, and we have no direct comparisons of different strategies in large numbers of patients with clinical events as outcomes. Even combined, randomised trials of these strategies had only four clinical events, and only the surrogate marker of endoscopic ulcer supports efficacy. Because we have two direct comparisons of coxib with NSAID plus PPI in two randomised trials in high-risk patients immediately after healing of a previous ulcer, and with no statistically significant difference, we can probably assume at least some degree of upper gastrointestinal protection with PPI use, although conclusions from these trials may not apply to those without a previous ulcer. For H2A, no such assumption is justified. We have evidence from one large randomised trial and two cohort studies that coxibs cause fewer problems in the lower bowel than NSAID and PPI. Use of NSAID plus PPI, and especially H2A, therefore fall some way short in terms of gastrointestinal protection.

The evidence from observational studies is that most patients at higher risk of gastrointestinal problems with NSAIDs, by virtue of age or previous history, are not usually given gastroprotection. Most of the studies relate to the period 2000–2002, and it may be that the introduction of better guidance since then has led to a change, but we could not find evidence of that. Two further studies published in 2006 confirm low use of gastroprotection in patients with gastrointestinal risk factors taking NSAIDs. One in primary care [54] reported a large uptake of coxibs over 1998–2002, and low use of gastroprotection with NSAIDs at any time over that period in patients aged 65 years or older. The second [55] surveyed prescribing by rheumatology specialists in 2003/4, and found that even in patients with four risk factors for gastrointestinal ulceration, gastroprotection was co-prescribed with NSAIDs in no more than 40% of patients.

The evidence we have about prescribing is that guidance has not generally been followed: three quarters of patients with at least one gastrointestinal risk factor did not receive a prescription for a gastroprotective agent. In individual studies in primary care adherence prescribing guidelines varied from 9% to 27% (Figure 1). It should be emphasised that this observational evidence is substantial, based on almost 1.6 million patients, over 900,000 of whom were prescribed NSAIDs, and studies often specifically tested adherence to evidence-based guidelines (as in over 700,000 patients [44]).

Patient adherence to prescribed gastroprotection was described in only one study [36], which found that adherence to NSAID plus PPI or H2A declined rapidly, so that after about six months the majority of patients were not taking the gastroprotection prescribed. This is in accord with many other studies of adherence to medicines. Adherence to low dose aspirin was only 47% after a year [56], was as low as 33% for statin and antihypertensive medicines when prescribed together [57], and was even about 20% with immunosuppressants following renal transplantation [58]. Coxibs cause less gastrointestinal harm, thus limiting or eliminating the need for co-prescription and the problem of adherence.

We chose not to extend this review beyond the narrow bound of gastroprotection, but other issues are important. For instance, NSAID and coxib clinical trials have a range of common adverse events [25]. Other serious adverse events, like cardiovascular problems, have to be considered, and it seems increasingly likely that these will be related to individual NSAIDs or coxibs rather than coxibs versus NSAIDs [12,13]. Long-term acid suppression itself may be associated with adverse events, including hip fracture [59] and vertebral fracture [60]. Patient choice is an increasingly important issue, and interestingly no experienced patient with knee osteoarthritis chose conventional NSAID therapy when presented with information about common therapies [61].

In this review the use of observational studies to evaluate adherence to clinical guidelines is not a limitation: the observational studies report what is happening in clinical practice. Moreover, many of these observational studies were large and inclusive, minimising potential for bias. Evidence from clinical trials (randomised, and controlled) and clinical practice (observational studies) tend to be similar when criteria of quality, validity and size are met [60]. It is seen for cardiovascular effects of coxibs and NSAIDs in meta-analyses of clinical trials [12] and observational studies [13].

For a strategy to be effective, it has to translate efficacy from clinical trials to clinical practice. With NSAIDs, but not coxibs, gastrointestinal protection can be delivered only if those patients who need gastroprotection are given it, and those who are given gastroprotection take it. Effectiveness evidence is summarised for the different strategies is in Table 4. The evidence for efficacy of co-prescription of gastroprotective agents in protecting the upper gastrointestinal tract is largely limited to the surrogate measure of endoscopic ulcers, though it is probable that bleeding events would be reduced. Such evidence we have suggests that PPIs have no efficacy in protecting the small bowel. From clinical practice, the evidence is that those patients who need gastroprotection do not get it, while those who get it do not take it. As a strategy, this is a considerable way short of ideal.

## Conclusion

There is considerable evidence that patients who need gastroprotection because they have at least one gastrointestinal risk factor do not get it, despite clear guidelines suggesting that they should. There is limited evidence that those who get gastroprotection in the form of PPI or H2A do not take it. As a strategy, this is a considerable way short of ideal.

## Abbreviations

PPI: proton pump inhibitor

H2A: histamine-2 receptor antagonist

NSAID: non-steroidal anti-inflammatory drug

Coxib: cyclooxygenase-2 selective inhibitor

## Competing interests

RAM, HJM and CP have received consulting and/or lecture fees from pharmaceutical companies and other organisations. The authors have received research support from charities and government sources at various times. No author has any direct stock holding in any pharmaceutical company.

## Authors' contributions

RAM was involved with the original concept, planning the study, data extraction, analysis, and preparing a manuscript; SD with data extraction, analysis, and writing; CP and HJM with writing and advice. All authors read and approved the final manuscript.

## Pre-publication history

The pre-publication history for this paper can be accessed here:



## Figures and Tables

**Table 1:** Summary of gastrointestinal evidence of efficacy of coxibs

**Reference**	**Study design**	**Population**	**Main outcomes**	**Main results**	**Relative risk, coxib compared with NSAID (95% CI)**
**Large randomised trials**					
Bombardier et al. N Engl J Med 2000 343: 1520–1528 [17]	Randomised trial powered for PUB outcome, comparing 50 mg rofecoxib with 1000 mg naproxen daily	Patients with RA, at least 50 years n = 8,076	Confirmed clinical upper GI events (perforation, bleeding symptomatic ulcer)	177 events, 53 complicated Confirmed events at 2.1/100 pt years with rofecoxib, 4.5 with naproxen Complicated 0.6 and 1.4 per 100 pt years	0.5 (0.3 to 0.6) 0.4 (0.2 to 0.8)
Silverstein et al. JAMA 2000 284: 1247–1255 [18]	Randomised trial powered for PUB outcome, comparing 800 mg celecoxib with 2400 mg ibuprofen and 150 mg diclofenac daily	Patients with OA or RA, ≥ 18 years n = 8,059	Confirmed upper GI ulcers and complication (bleeding, perforation, obstruction)	83 events including symptomatic ulcers, 35 complicated Confirmed events at 2.1/100 pt years with celecoxib, 3.5 with NSAID Complicated at 0.8 and 1.5 per 100 pt years	0.6 (0.4 to 0.9) 0.5 (0.3 to 1.1)
Schnitzer et al. Lancet 2004 364: 665–674 [19]	Randomised trial powered for PUB outcome, comparing 400 mg lumiracoxib with 2400 mg ibuprofen and 1000 mg naproxen daily	Patients with OA, at least 50 years n = 18,325	Confirmed upper GI ulcers and complication (bleeding, perforation, obstruction)	283 events, 112 complicated Confirmed events at 1.0% with lumiracoxib, 1.5% with NSAID Complicated events at 0.3% with lumiracoxib, 0.9% with NSAID	0.7 (0.5 to 0.8) 0.3 (0.2 to 0.5)

**Meta-analyses of randomised trials**					
Langman et al. JAMA 1999 282: 1929–1933 [20]	Presecified meta-analysis of eight randomised trials of rofecoxib versus NSAIDs	OA patients, mean age 63 years n = 5,435	Confirmed clinical upper GI events (perforation, bleed, ulcer)	35 confirmed complicated events Complicated events at 1.3/100 pt years with rofecoxib, 2.6 with NSAID	0.5 (0.3 to 1.0)
Goldstein et al. Am J Gastroenterol 2000 95: 1681–1690 [21]	Meta-analysis of 14 randomised trials of celecoxib versus NSAIDs	OA or RA patients, mean age 60 years n = 11,008	Confirmed clinical upper GI events (perforation, bleed, ulcer)	11 confirmed complicated events Complicated events at 0.2/100 pt years with celecoxib, 1.7 with NSAID	0.2 (0.1 to 0.5)
Edwards et al. Pain 2004 111: 286–296 [22]	Meta-analysis of nine randomised trials of valdecoxib versus NSAIDs	OA or RA patients, n = 5,726	Clinically significant upper GI bleed	10 confirmed complicated events Complicated event rate 0.1% with valdecoxib, 0.4% with NSAID	0.2 (0.04 to 0.8)
Goldstein et al. Aliment Pharmacol Ther 2004 20: 527–538 [23]	Meta-analysis of eight randomised trials of valdecoxib versus NSAID	OA or RA patients, mean age 58 years n = 7,434	Confirmed clinical upper GI events (perforation, bleed, ulcer)	88 symptomatic ulcers, 19 complicated Symptomatic + complicated 0.8% with valdecoxib, 3.3% with NSAID Complicated 0.2% with valdecoxib, 0.5% with NSAID	0.3 (0.2 to 0.4) 0.4 (0.1 to 0.9)
Hooper et al. BMJ 2004 329: 948–952 [24]	Meta-analysis of 17 randomised trials of coxibs versus NSAIDs	n = 25,564	Variety of outcomes reported, including serious gastrointestinal complications, and symptomatic ulcers	114 serious gastrointestinal complications, 0.36% with coxib, 0.73% with NSAID 288 symptomatic ulcers, 0.8% with coxib, 1.8% with NSAID	0.5 (0.4 to 0.8) 0.5 (0.4 to 0.6)
Moore et al. Arth Res Ther 2005 7:R644–R655 [25]	Meta-analysis of 31 randomised trials of celecoxib versus NSAIDs	OA or RA n = 39,605 (31,171 in analysis of ulcers and bleeds)	Variety of outcomes reported including clinical ulcers and bleeds	184 clinical ulcers or bleeds, 0.4% with celecoxib, 0.9% with NSAID	0.6 (0.5 to 0.8)

**Large observational studies**					
Mamdani et al. BMJ 2002 325: 624–630 [26]	Observational cohort study	Users of NSAID, coxib, or non users. Total population about 144,000	Hospital admission for upper gastrointestinal bleeding	82 events with controls, 17 with NSAID, 75 with coxib Rofecoxib, but not celecoxib had significantly greater association with bleeding than controls	Celecoxib compared with NSAID 0.2 (0.1 to 0.4) Rofecoxib compared with NSAID 0.5 (0.3 to 1.0)
MacDonald et al. Gut 2003 52: 1265–1270 [27]	Retrospective cohort analysis	Users of NSAID, coxibs, and non users. Total 26,000 incident cases of upper gastrointestinal haemorrhage	Hospital admission for upper gastrointestinal bleeding in high risk patients	2,875 events on NSAID, 4 on coxib Adjusted relative risk	0.4 (0.1 to 1.0)
Norgard et al. Aliment Pharmacol Ther 2004 19: 817–825 [28]	Population based case-control study	Users of NSAID, coxibs, and non users. 780 incident cases in patients with high risk of gastrointestinal bleeding	Hospital admission for upper gastrointestinal bleeding	35 patients had been exposed to coxib (4.5%) 97 patients had been exposed to NSAID (12%) Rofecoxib, but not celecoxib had significantly greater association with bleeding than controls	0.4 (0.3 to 0.5)

**Table 2:** Number of events on which overall conclusions about the efficacy of gastrointestinal protection strategies were based in a systematic review [24]

	**Number of events recorded**		
	
**Outcome**	**Coxib**	**NSAID + PPI**	**NSAID + H2A**
Serious gastrointestinal complications	**226**	3	1
Symptomatic ulcers	**452**	18	1
Endoscopic ulcer	**522**	**281**	**250**
Anaemia	**464**	none	1
Withdrawal due to gastrointestinal symptoms	**2171**	48	57

Data from Hooper et al, 2004 [24]. The numbers show the actual numbers of events reported for each outcome in the paper, and the bold numbers indicate those where event rates were significantly lower with the strategy used than with NSAID alone

**Table 3:** Summary of gastroprotection in clinical practice

**Reference**	**Study design**	**Population**	**Main outcomes**	**Main results**
**Adherence and appropriateness of gastroprotection prescribing**				
Sturkenboom et al. Aliment Pharm Ther 2003 18: 1137–1147 Holland [36]	Retrospective cohort study using primary care database between 1997 and 2003	Patients aged ≥18 years with 12 months data in database (382,000 patients; 80,000 users of NSAIDs)	Adherence to gastroprotective agents	Of 65,190 patients taking NSAIDs, 784 had PPI or H2A, in about equal numbers. Patients prescribed gastroprotection were significantly older, had more risk factors, and had more cardiovascular disease 85% of H2A prescriptions were below recommended dose 31% of patients receiving PPI were non-adherent intially, but only about 40% took PPI long term

**Appropriateness of gastroprotection prescribing**				
Smalley et al. Arthritis Rheum 2002 46: 2195–2200 USA [37]	Retrospective cohort study using Medicaid database during 1999–2000	Patients aged ≥ 50 years, with 12 months data, filled one NSAID prescription (319,000, of whom 107,000 received at least one NSAID prescription)	Frequency of use of gastroprotective measures according to NSAID use and risk factors	Recommended gastroprotection in 9% of patients with one risk factor, 11% of those with two risk factors. Most patients had no gastroprotection, whilst about 25% had inadequate gastroprotection. No information about adherence
Pilotto et al. Drugs Aging 2003 20:701–710 Italy [38]	Prospective study of drug use by patients aged ≥ 65 years. 3,200 patients of 63 randomly chosen general practitioners, in 1999	Patients aged ≥ 65 years Of 3,200 patients, 800 prescribed NSAID	Use of prescribed medicines	NSAID and high-dose aspirin prescribed for 25% of patients Use of GI protective drugs was 24% of NSAID users with at least one risk factor by virtue of age, slightly higher than for non users of NSAIDs No information about adherence
Sturkenboom et al. Rheumatology 2003 42 (Suppl 3):iii23–iii31 Holland [39]	Retrospective cohort study using primary care database between 1997 and 2002	Patients aged ≥18 years with 12 months data in database (382,000 patients; 80,000 users of NSAIDs)	Prevalence of prophylactic gastro-protective strategies, and association with risk factors	In patients with at least one risk factor, 87% had no gastroprotective strategy Proportion with no gastroprotection reduced over time No information about adherence
Hartnell et al, Am J Geriatr Pharmacother 2004 2: 171–180 Canada [40]	Retrospective cross-sectional study of pharmacy database for older people, 2001–2002	Patients aged ≥ 65 years with 12 months data who filled prescription for NSAID, coxib, or high-dose aspirin (14,600 older patients using NSAID or coxib)	Use of gastroprotective strategies	Of 11,000 NSAID users, 14% received gastroprotection Of 3,600 coxib users, 5% received gastroprotection Gastroprotection not used in 65% NSAID alone used in 67% of patients with only age as a risk factor, falling to 63% with one additional, and 52% with two additional risk factors No information about adherence
Dominick et al. Ann Pharmacother 2004 38: 1159–1164 USA [41]	Retrospective cohort study of sample of 4,338 veterans with GI bleeding in 1999	Patients had ICD code for GI ulceration or bleeding. Veterans were predominantly male, 50% aged 65 years or older	Use of gastroprotective strategies and prescribing NSAIDs in six months following event	1% prescribed coxib 20% prescribed NSAID; of these 75% prescribed gastroprotection, 25% no gastroprotection No information about adherence
Herings & Goettsch. Ann Pharmacother 2004 38: 760–763 Holland [42]	Nested case control analysis of database (1 million people; 10,000 patients included), 2000 to 2001	Patients had to have at least two prescriptions for NSAID, with total duration > 100 days	Adequate gastroprotection (> 400 μg misoprostol; ≥ 2 times recommended dose of H2A; ≥ 1 times recommended dose of PPI)	One or more gastroprotective strategies used in 43% of NSAID users. Of these 65% were adequate, and 35% inadequate Use of gastroprotection was linked to having 2 or more risk factors, history of ulcers, and older age 25% of NSAID users were also taking anticoagulants, corticosteroids, or low dose aspirin No information about adherence
Sebaldt et al. Am J Manag Care 2004 10:742–750 Canada [43]	Cross-sectional study of 5,459 patients of 119 physicians	Primary care physicians with hgih volume NSAID prescribing practices. OA patients had to be prescribed an NSAID	Adherence to appropriate prescribing of coxibs and NSAIDs, with or without gastroprotection	In patients with no GI risk factors (39% of total), 33% of prescribing was appropriate In patients with at least one GI risk factor (61% of total), 74% of prescribing was appropriate More use of coxibs with prior bleed, more severe pain, or with concomitant warfarin
Abraham et al. Gastroenterol 2005 129: 1171–1178 USA [44]	Cross-sectional study of database, linked to other files (707,000 NSAID users, 303,000 high risk patients), 2002	Various definitions of high gastrointestinal risk, including age ≥ 65 years	Adherence to gastroprotection guidelines	43% of NSAID users were at high risk of GI complications 73% of these not prescribed gastroprotection 27% of these prescribed gastroprotection (18% NSAID plus PPI, 9% coxib) Greater gastroprotection use with two or more risk factors Predictors of gastroprotection were previous upper gastrointestinal event, anticoagulant use, aspirin use, rheumatological disease
Thompson et al. Rheumatology 2005 44:1308–1310 UK [45]	Cross-sectional survey of primary care practice 7,598 patients in practice in total	267 patients receiving repeat prescriptions for coxib or NSAID 204 NSAID 63 coxib	Prescribing according to NICE guidance	69% NSAID users had one or more GI risk factors; antacids prescribed in 24% of those with a risk factor 74% coxib users had one or more GI risk factors; antacids prescribed in 6% of those with a risk factor
Price-Forbes et al. Rheumatology 2005 44:921–924 UK [46]	Questionnaire survey of all patients attending clinics in 18 rheumatology units over 2 weeks	2,846 patients, of whom 791 were taking NSAIDs and 373 coxibs. 65% of users had diagnosis of OA or RA	Prescribing according to GI risk factors	Of NSAID users, 92% had at least one GI risk factor (mostly prolonged use, and age ≥ 65 years); only 8% received appropriate treatment. Gastroprotective drug prescribed for 191 patients (24%), of which 56% were PPI Of coxib users, 97% of prescribing was appropriate, with 77 (21%) taking gastroprotective drug
Pilotto et al. Aliment Pharmacol Ther 2005 22: 147–155 Italy [[47]	Prospective study of drug use by patients aged ≥ 65 years. 5,500 patients of 133 general practitioners, in 2003	Patients aged ≥ 65 years	Use of prescribed NSAIDs, and GI symptoms	NSAID use in 6% Coxib use in 3% New prescriptions for drugs for acid-related disorders in 13% of NSAID users, 6% of coxib users No information on adherence

**General information about gastroprotection prescribing**				
Schnitzer et al. Clin Ther 2001 23: 1984–1998 USA [48]	Retrospective analysis of prescription database for 1998. 3 million new users of NSAIDs	At least one NSAID prescription during 1998, and no use in prior 120 days (< 30 days acute; > 30 days chronic); 34% of chronic users 60 years or older	Use of gastroprotective medicines, by NSAID	In 1.4 million chronic users, the mean prescription was for 67 days supply. Gastroprotection was prescribed for 14%, covering 22% of NSAID days No information about risk factors or adherence
Teeling et al. Br J Clin Pharmacol 2004 57: 337–343 Ireland [49]	Retrospective analysis of prescription database, 2000–2001 (1.2 million people). About 25,000 NSAID/coxib users	Patients aged 16 years or older prescribed an NSAID	Use of gastroprotective medicines, by NSAID	No gastroprotection in about 80%. PPI used in about 15%. Use of PPI higher with coxibs than with non-selective NSAIDs. Coxibs much more likely to be prescribed in over 65s. No information about risk factors or adherence
Pilotto et al. Aliment Pharmacol Ther 2005 22: 147–155 Italy [47]	Prospective study of drug use by patients aged ≥ 65 years. 5,500 patients of 133 general practitioners, in 2003	Patients aged ≥ 65 years	Use of prescribed NSAIDS, and GI symptoms	Non-selective NSAID use in 6% Coxibs use in 3% New prescriptions of drugs for acid-related disorders in 13% of NSAID users, 6% of coxib users No information about adherence

**Table 4:** Summary of effectiveness

**Strategy**	**Efficacy**	**Appropriate prescribing**	**Adherence**
**Upper gastrointestinal injury**			
Coxib	Extensive, robust evidence	Low	Effective
NSAID + PPI	Surrogate only	Low	Low adherence
NSAID + H2A	Surrogate only	Low	Low adherence
**Lower gastrointestinal injury**			
Coxib	Some evidence of efficacy	Low	Effective
NSAID + PPI	Some evidence of lack of efficacy	Low	Low adherence
NSAID + H2A	No evidence	Low	Low adherence

Summarised from Hooper et al, 2004 [24].
